# A Case of Sigmoid Colon Cancer with Patent Foramen Ovale and Left-Sided Inferior Vena Cava Initially Detected during the Workup for Cerebral Infarction

**DOI:** 10.70352/scrj.cr.26-0043

**Published:** 2026-04-17

**Authors:** Yusuke Yoshida, Yuki Matsumi, Ryohei Shoji, Toshiyoshi Fujiwara

**Affiliations:** Department of Gastroenterological Surgery, Okayama University Graduate School of Medicine, Dentistry and Pharmaceutical Sciences, Okayama, Okayama, Japan

**Keywords:** patent foramen ovale, left-sided inferior vena cava, laparoscopic sigmoidectomy, paradoxical embolism

## Abstract

**INTRODUCTION:**

Patent foramen ovale (PFO) is present in approximately 25% of adults, whereas a left-sided inferior vena cava (IVC) is a rare congenital vascular anomaly. Both conditions are associated with thromboembolic risk; however, their coexistence in patients with colorectal cancer has not been reported. We report a rare case of sigmoid colon cancer complicated by PFO and left-sided IVC, initially detected during the diagnostic workup for cerebral infarction.

**CASE PRESENTATION:**

A 69-year-old man was diagnosed with cerebral infarction, and transthoracic echocardiography revealed PFO with a positive bubble test. While receiving anticoagulation therapy, he developed persistent hematochezia, and subsequent investigations revealed sigmoid colon cancer with a solitary liver metastasis. Contrast-enhanced CT demonstrated a left-sided IVC running along the left side of the abdominal aorta and joining the right renal vein. Laparoscopic sigmoidectomy with D3 lymph node dissection was safely performed with careful attention to the aberrant venous anatomy. The postoperative course was uneventful, and anticoagulation therapy was resumed early. The patient subsequently underwent hepatic resection after systemic chemotherapy.

**CONCLUSIONS:**

This case highlights the importance of accurate preoperative anatomical evaluation and careful perioperative thromboembolic management in laparoscopic colorectal surgery involving coexisting PFO and major vascular anomalies.

## Abbreviations

IVC
: inferior vena cava
PFO
: patent foramen ovale

## INTRODUCTION

PFO is present in approximately 25% of adults,^1–3)^ while a left-sided IVC is a rare congenital vascular anomaly with an incidence of 0.2%–0.5%.^4,5)^ Both conditions are known to be associated with an increased risk of thromboembolic events.^3,6–9)^ However, to the best of our knowledge, the coexistence of PFO and left-sided IVC in a patient with colorectal cancer has not been previously reported. Herein, we report a rare case of sigmoid colon cancer complicated by PFO and left-sided IVC, which was initially detected during the diagnostic workup for cerebral infarction. This case highlights the importance of thorough preoperative anatomical assessment in laparoscopic colorectal surgery and careful perioperative thromboembolic management.

## CASE PRESENTATION

**Fig. 1:**
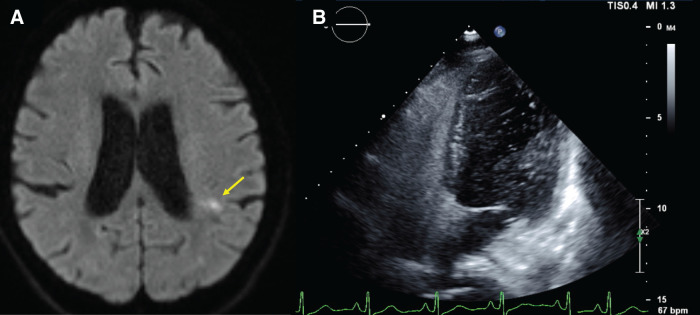
Neuroimaging and cardiac findings. (**A**) Diffusion-weighted MRI of the brain showing an acute cerebral infarction (arrow). (**B**) Transthoracic echocardiography with a bubble test demonstrating a positive right-to-left shunt at rest (Grade 3), consistent with PFO. PFO, patent foramen ovale

**Fig. 2:**
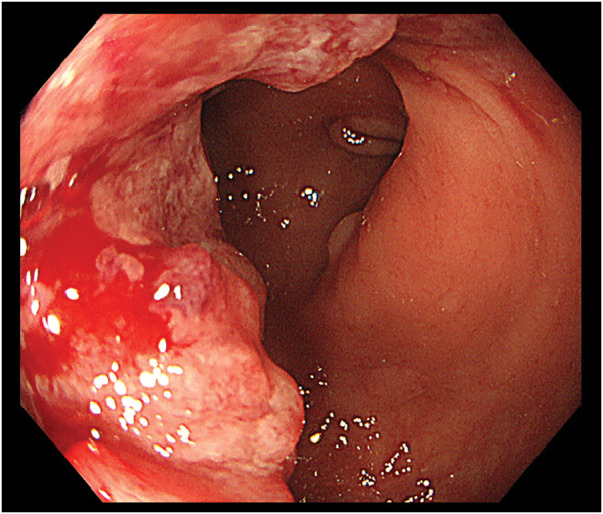
Colonoscopic findings. Colonoscopy revealed a semi-circumferential type 2 tumor in the sigmoid colon.

**Fig. 3:**
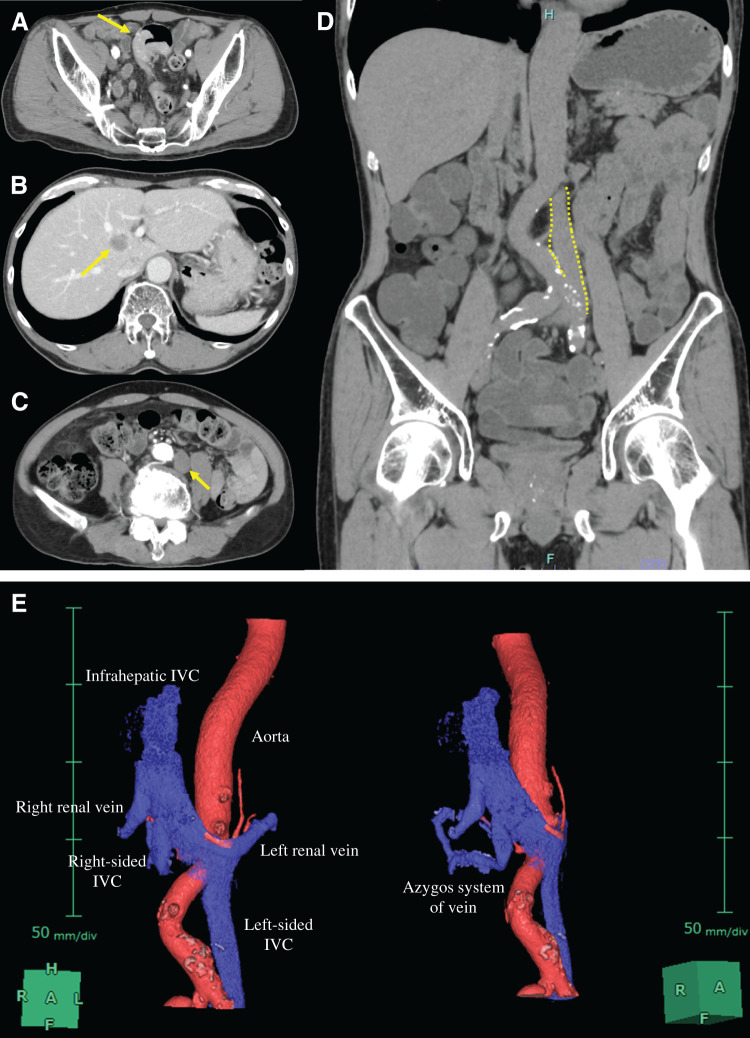
Contrast-enhanced CT findings. (**A**) Contrast-enhanced CT showing contrast-enhancing wall thickening of the sigmoid colon (arrow). (**B**) A solitary metastatic lesion was observed in the right hepatic lobe (arrow). (**C**, **D**) CT demonstrating a left-sided IVC (**C**: arrow; **D**: dotted line) running along the left side of the abdominal aorta. (**E**) 3D CT reconstruction demonstrating a left-sided IVC with azygos/hemiazygos continuation. The IVC runs along the left side of the abdominal aorta, joins the left renal vein, and continues as the infrahepatic IVC. This reconstruction clearly illustrates the anomalous venous anatomy and its spatial relationship to adjacent structures. IVC, inferior vena cava

**Fig. 4:**
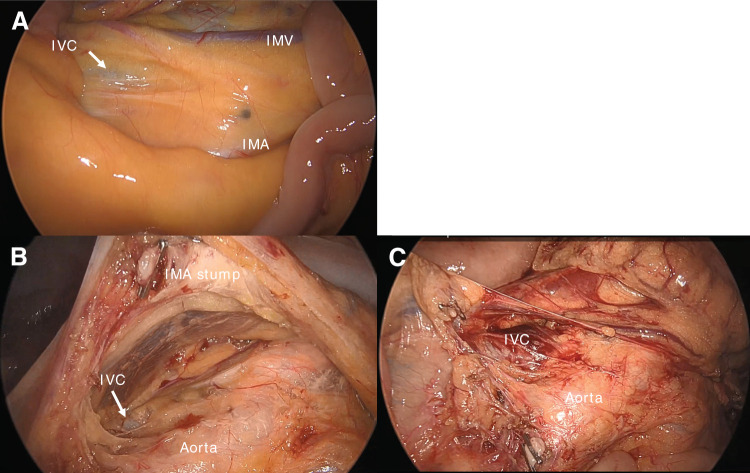
Intraoperative findings. (**A**, **B**) Intraoperative view showing the left-sided IVC (arrow) running immediately dorsal to the left mesocolon during medial-to-lateral dissection. (**C**) Operative field after completion of D3 lymph node dissection without vascular injury. IMA, inferior mesenteric artery; IMV, inferior mesenteric vein; IVC, inferior vena cava

**Fig. 5:**
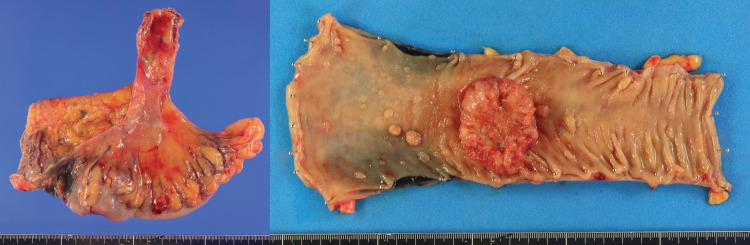
Resected specimen. Macroscopic appearance of the resected sigmoid colon specimen showing a type 2 tumor.

A 69-year-old man presented to another hospital with acute cognitive dysfunction after waking, including difficulty operating household appliances and a mobile phone. He was diagnosed with cerebral infarction (**Fig. 1A**). His medical history was significant for hypertension. His neurological symptoms improved following treatment with ozagrel sodium and edaravone. Transthoracic echocardiography with a bubble test revealed a positive result at rest (Grade 3) (**Fig. 1B**), leading to the diagnosis of PFO. Transcatheter closure was proposed; however, the patient declined, and anticoagulation therapy with edoxaban was continued. After discharge, the patient developed persistent hematochezia while receiving anticoagulation therapy, which prompted further investigation. Colonoscopy revealed sigmoid colon cancer. Contrast-enhanced abdominal CT demonstrated a solitary liver metastasis, and the patient was referred to our institution with suspected Trousseau syndrome. On admission, laboratory findings showed mild anemia and elevated tumor markers, while coagulation parameters were within normal limits (**Table 1**). Colonoscopy revealed a semi-circumferential type 2 lesion in the sigmoid colon (**Fig. 2**), and biopsy confirmed moderately differentiated tubular adenocarcinoma. Contrast-enhanced CT showed contrast-enhancing wall thickening of the sigmoid colon (**Fig. 3A**), enlarged intermediate lymph nodes suspicious for metastasis, and a solitary metastasis in the right hepatic lobe (**Fig. 3B**). Additionally, a left-sided IVC was identified (**Fig. 3C** and **3D**), running along the left side of the abdominal aorta and joining the left renal vein to form the normal infrahepatic IVC (**Fig. 3E**). This anomaly was classified as a left-sided IVC with azygos/hemiazygos continuation.^5)^ Given the presence of symptoms from the primary tumor, laparoscopic sigmoid colectomy with D3 lymph node dissection was performed prior to systemic therapy. Intraoperatively, the IVC was observed running immediately dorsal to the left mesocolon, instead of its usual right-sided position (**Fig. 4A**). This anatomical variation altered the usual surgical landmarks during the medial-to-lateral approach. In standard anatomy, the ureter and gonadal vessels serve as key retroperitoneal landmarks during medial mobilization. However, in this case, the left-sided IVC was located dorsal to the left mesocolon and could potentially be encountered before identifying the ureter and gonadal vessels. Therefore, particular attention was paid to maintaining the correct embryological dissection plane along the dorsal surface of the mesocolon before exposing the retroperitoneal structures (**Fig. 4B**). By carefully preserving the retroperitoneal layer and avoiding deep dissection until the anatomical relationships were fully confirmed, vascular injury was successfully prevented (**Fig. 4C**). The operative time was 2 h and 26 min, and blood loss was minimal. The postoperative course was uneventful, and the patient was discharged on POD 8. **Fig. 5** shows the resected specimen. Postoperative chemotherapy with mFOLFOX6 plus panitumumab was initiated. Four months after colectomy, the patient underwent hepatic resection for the metastatic liver tumor. Anticoagulation therapy was also resumed after subsequent liver resection without thromboembolic complications. He has since been followed without additional chemotherapy.

**Table 1:** Laboratory findings on admission

WBC (×10^3^/μL)	5.58	Na (mmol/L)	139	AST (U/L)	15
RBC (×10^6^/μL)	3.63	K (mmol/L)	5.0	ALT (U/L)	11
Hb (g/dL)	11.6	Cl (mmol/L)	104	ALP (U/L)	122
Ht (%)	37.4	Ca (mg/dL)	9.2	γ-GTP (U/L)	13
Plt (×10^3^/μL)	390	UN (mg/dL)	12.3	LDH (U/L)	225
PT (s)	10.5	Cr (mg/dL)	0.90	CEA (ng/mL)	6.64
PT (%)	122	TP (g/dL)	6.9	CA19-9 (U/mL)	119.6
PT-INR	0.92	Alb (g/dL)	4.0		
APTT (s)	26.2	T-Bil (mg/dL)	0.54		
D-dimer (μg/mL)	<0.5	D-Bil (mg/dL)	0.04		

Mild anemia and elevated tumor markers (CEA and CA19-9) were observed, whereas coagulation parameters were within normal ranges. D-dimer was below the detectable limit.

Alb, albumin; ALP, alkaline phosphatase; ALT, alanine transaminase; APTT, activated partial thromboplastin time; AST, aspartate transaminase; CA19-9, carbohydrate antigen 19-9; CEA, carcinoembryonic antigen; Cr, creatinine; D-Bil, direct bilirubin; Hb, hemoglobin; Ht, hematocrit; INR, International Normalized Ratio; LDH, lactate dehydrogenase; Plt, platelet; RBC, red blood cell; T-bil, total bilirubin; TP, total protein; UN, urea nitrogen; WBC, white blood cell; γ-GTP, glutamyl transferase

## DISCUSSION

Although Trousseau syndrome was initially suspected at the referring hospital, the patient had stage IVa disease with a solitary liver metastasis, preserved coagulation function, and relatively good general condition. Furthermore, the patient’s D-dimer level at the onset of cerebral infarction was 0.8 μg/mL, which was within the normal range. Cancer-associated hypercoagulability, including Trousseau syndrome, is often accompanied by elevated D-dimer levels.^10)^ Although tumor-related thrombosis cannot be completely excluded based solely on D-dimer levels, these findings made Trousseau syndrome less likely in the present case. The cerebral infarction was considered to be a paradoxical embolism in which venous thrombosis, which may be associated with altered venous flow in patients with left-sided IVC, passed into the cerebral arterial circulation through the PFO rather than the pulmonary circulation. Trousseau syndrome is relatively uncommon in colorectal cancer,^11,12)^ and the absence of multiple or recurrent cerebral infarctions further supported the conclusion that Trousseau syndrome was unlikely in this case.^10–12)^

Although no specific perioperative management guidelines for patients with PFO have been established, previous studies suggest an increased risk of perioperative ischemic stroke in this population.^13,14)^ Therefore, patients with PFO should be considered at high thromboembolic risk during noncardiac surgery.

Preoperative evaluation for venous thromboembolism, including contrast-enhanced CT or lower-extremity ultrasonography, may be warranted. Perioperative mechanical prophylaxis, such as intermittent pneumatic compression or compression stockings, along with early ambulation, is essential to minimize venous stasis.^15)^ Routine placement of an IVC filter is not recommended in the absence of documented deep vein thrombosis.^15)^ Furthermore, appropriate and timely resumption of anticoagulation therapy is crucial, balancing thrombotic and bleeding risks.^15)^

In the present case, anticoagulation therapy with a direct oral anticoagulant was initiated preoperatively and resumed promptly on POD 2. Preoperative contrast-enhanced CT revealed no evidence of venous thrombus; therefore, an IVC filter was not placed. The anomaly was classified as a left-sided IVC with azygos/hemiazygos continuation.^5)^ Although routine IVC filter placement is not recommended in the absence of documented thrombosis, the presence of a left-sided IVC may pose technical challenges for filter placement and should be carefully considered if such intervention is contemplated.^5)^ Preoperative recognition of this venous anomaly is therefore essential not only for safe surgical dissection but also for planning potential endovascular interventions.

Intermittent pneumatic compression was applied intraoperatively and postoperatively, and early ambulation was encouraged after surgery.

Another important consideration is whether preoperative PFO closure could have further reduced perioperative risk. Closure of a PFO may theoretically decrease the risk of paradoxical embolism and prevent systemic embolization of microbubbles that would otherwise be filtered in the pulmonary circulation.^16)^ In addition, it may simplify perioperative management by allowing standard venous thromboembolism prophylaxis without concern for right-to-left shunting. However, there is currently no established consensus regarding routine preoperative PFO closure before noncardiac surgery.^13)^ In the present case, PFO closure was not performed at the patient’s request, and careful perioperative thromboembolic prophylaxis was adopted instead.

Even in cases with a left-sided IVC, adherence to the standard medial approach based on correct anatomical landmarks allows safe dissection. The key is to remain ventral to the ureterohypogastric nerve fascia while staying just dorsal to the mesocolon. In particular, conscious recognition of the posterior surface of the mesocolon helps maintain the correct anatomical layer and prevents unnecessary entry into the retroperitoneal space. By consistently following this plane, the left-sided IVC can be safely preserved dorsally. In the present case, the tumor was located in the sigmoid colon, and cranial dissection of the left mesocolon was limited. Standard laparoscopic surgery provided sufficient visualization to safely identify the anomalous anatomy, and 3D or robot-assisted surgery was not considered mandatory. However, if the lesion had been located in the descending colon or at the splenic flexure, more extensive cranial mobilization would have been required, necessitating greater caution regarding the course of the left-sided IVC. Similarly, cases requiring para-aortic lymph node dissection or presenting with more complex vascular variations may benefit from 3D or robotic-assisted surgery to facilitate safer dissection.

Postoperative treatment decisions were primarily guided by oncological principles. As no active venous thrombosis was identified and anticoagulation was appropriately managed, standard therapy was administered. Given the RAS wild-type status, an anti-EGFR antibody was selected. No thromboembolic events occurred during follow-up.

## CONCLUSIONS

Sigmoid colon cancer complicated by both PFO and left-sided IVC is extremely rare. In this case, preoperative recognition of the vascular anomalies allowed the procedure to be performed safely using a standard laparoscopic approach. Additionally, early resumption of anticoagulation therapy was achieved based on an assessment of perioperative thromboembolic risk. This case underscores the importance of preoperative anatomical evaluation and careful perioperative management in colorectal cancer surgery involving coexisting PFO and major vascular anomalies.

## DECLARATIONS

### Availability of data and materials

The data that support the findings of this study are available from the corresponding author upon reasonable request.
